# We Need to Change: Integrating Psychological Perspectives Into the Multilevel Perspective on Socio-Ecological Transformations

**DOI:** 10.3389/fpsyg.2021.655352

**Published:** 2021-04-26

**Authors:** Marlis C. Wullenkord, Karen R. S. Hamann

**Affiliations:** Social, Environmental, and Economic Psychology, Department of Psychology, University of Koblenz-Landau, Landau, Germany

**Keywords:** socio-ecological transformation, multilevel perspective, self-determination theory, self-efficacy theory, agency, socio-technical transitions, opinion, environmental psychology

## Psychology's Place in Socio-Technical Transition Research

“*By embedding humans into systemic models […] we can see that even when we are talking about global transformations, the source of intentional change is human thinking, feeling, and acting. Socio-ecological-technological systems are created, ordered, and stabilized through human decision-making and (often) conscious creation of regime structures.”*—Göpel (2016, p. 50/51)

It sounds self-evident when Göpel explains how deeply ingrained humans are in transition processes. However, when working on a virtual lecture series called *Psychologie des sozial-ökologischen Wandels* (*The Psychology of Socio-Ecological Change*)^1^ we felt challenged when attempting to connect all parts into a consistent narrative on the connections of psychology and a socio-ecological transformation (i.e., the deep transformation of society aiming for de-carbonization and socio-ecological justice, WBGU, 2011). With this challenge came ideas about transformation-oriented psychology that we feel inspired to share.

Previous research acknowledged that cultural worldviews and mindsets are essential for transformations (Meadows, 1999). The status quo and associated tangible structural outcomes are a result of human relationships and agency over time (Elias, 1982). Psychology as the study of human perception and behavior can contribute to transition research by investigating the processes underlying human agency. Nevertheless, psychological perspectives are rarely explicitly integrated into socio-technical transition research (Bögel and Upham, 2018). There have been pleas to clarify the role of individual-level processes in transitions (see Cattaneo et al., 2014; Bögel and Upham, 2018; Upham et al., 2020; Becker et al., 2021) and we personally experience a certain openness within psychology to do so. Kazdin (2009) even ascribes a crucial role to psychology in connecting different research areas. So why have those appeals only rarely been put into practice?

Environmental psychology historically focused on intra-individual factors (e.g., attitudes, control beliefs) and used them to explain pro-environmental behavior. As a result, it has been criticized for making somewhat mechanistic and reductionist assumptions, treating psychological constructs as isolated factors (see Dijk et al., 2016), and neglecting that contextual factors like larger-scale social structures and ecological processes influence behavioral outcomes (see Steg and Vlek, 2009). There are undeniable ontological and epistemological differences between psychological and transition science. Moreover, transformations are challenging to capture using psychological methods, and disciplinary research often earns more (career) credits than interdisciplinary research. Nonetheless, we believe that psychological perspectives are crucial for transformations but that transition researchers will only integrate psychological perspectives if they consider processes and outcomes relevant for understanding transformations. Placing one's own research into transition-oriented approaches may be a challenging first step due to the difficulties inherent in interdisciplinary work, given the context-specificity of both the research object and transition research itself, and because it remains unclear how to do so in a meaningful way. Nevertheless, we present an attempt to make psychological perspectives more impactful through theoretical integration, using two psychological theories and an exemplifying transition-model (see current debate by Nielsen et al., 2021 and Van Valkengoed et al., 2021).

### A Multilevel Approach to Transformations

There are several systemic models explaining socio-technical transitions, like the Multilevel Perspective (MLP, Geels and Schot, 2007, 2010) or the Multiphase Concept (Mersmann et al., 2014). One currently predominant model is the MLP (Geels and Schot, 2010). It looks at how socio-technical societal subsystems interact in transformation-processes across time and space: The landscape (macro-level; e.g., megatrends like climate change, the market system, hegemonic paradigms^2^), regimes (meso-level; e.g., policy, technology, science), and niches (micro-level). Higher levels—institutionalized, inertial, and historically rooted—are impactful but slow and difficult to change. Regimes are stabilized through path-dependencies like institutionalization or social-psychological infrastructures (e.g., norms, shared beliefs, see Welzer, 2011). They hamper individuals to imagine alternatives, lock the status quo, and prevent rapid change. Change occurs most readily in niches that provide safeguarded spaces to test radical socio-technical innovations. When regimes are destabilized, for instance because of landscape-level pressures like climate change, windows of opportunity open, and niche-innovations can establish themselves in regimes. While the MLP is useful for understanding socio-technical innovations, it is difficult to pinpoint human agency in it (see Geels, 2011 for a discussion; see Winner, 1986 for a fundamental critique of a technology-focus as lever of change).

Göpel (2016) explicitly acknowledges individuals and hegemonic paradigms in transformations by adding two layers: The mini-level contains individuals making up institutions. The meta-level represents the “hegemonic paradigm and common sense framework that serves as a reference for individual strategies and narratives” of change (p. 47). Both levels interact: The mini-level influences the meta-level because every individual contributes to changing and shaping the future paradigm and thereby reality. The meta-level is deeply embedded in the meso-, micro-, and mini-levels and mediates between them. For instance, it affects how individuals in specific regimes think (cognitive lock-ins, see Welzer, 2011).

## Theoretical Integration of Psychological Constructs Into GÖPEL'S MLP

**Figure 1:**
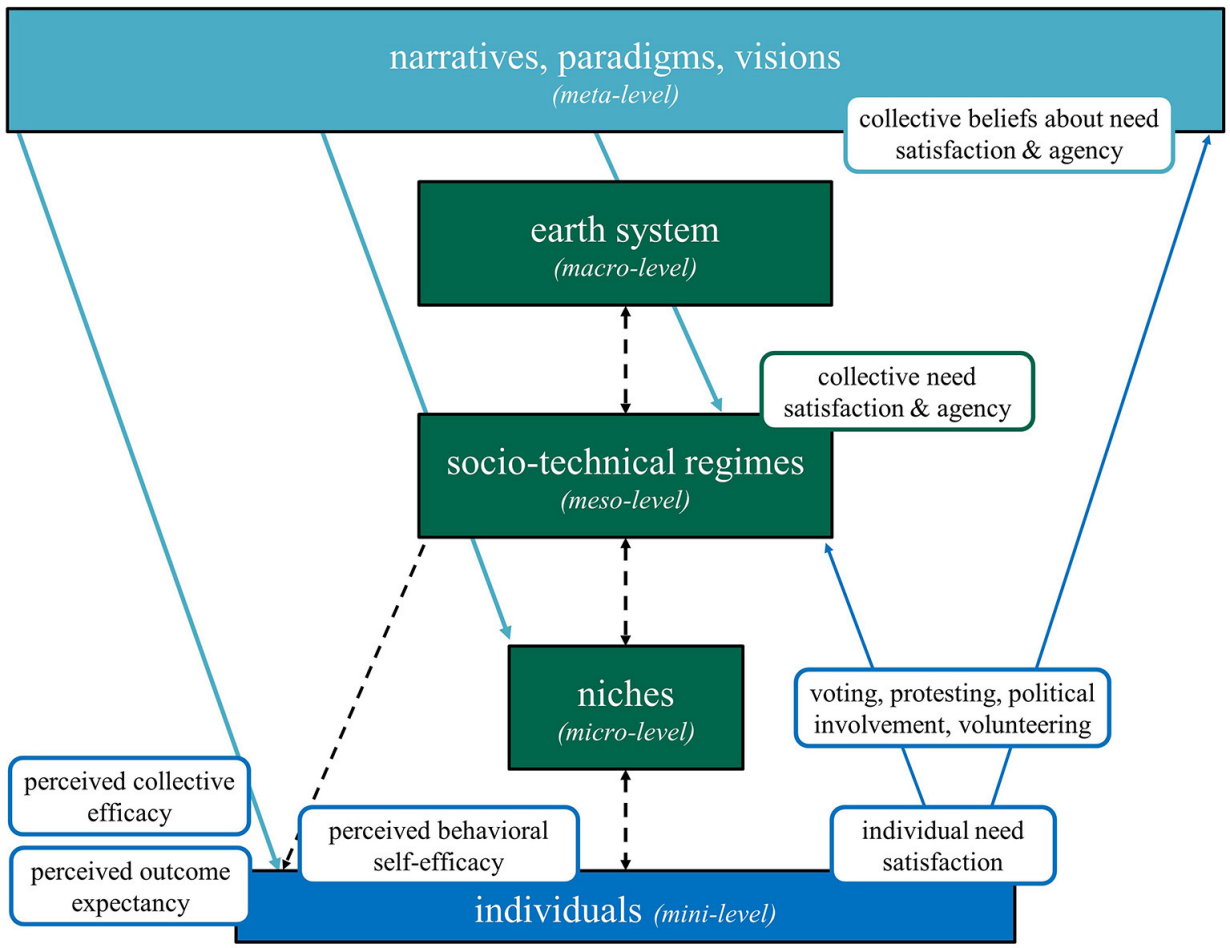
Göpel's extended MLP. Arrows represent interactions between levels. In rounded rectangles, we exemplarily embed basic premises of Self-Determination Theory and concepts related to Self-Efficacy Theory (graphic adapted from Göpel, 2016, p. 47). The meta and meso-level set structural boundaries to individual need satisfaction and agency, and affect psychological perceptions such as beliefs about need satisfaction, collective efficacy, outcome expectancy, and behavioral self-efficacy. Psychological outcomes such as individual need satisfaction feed back into higher levels, for instance via voting, protesting, political involvement or volunteering (in niches). Collective need-satisfaction and actual collective and motivation arising from efficacy beliefs agency might be located at the meso-level. Collective beliefs about need-satisfaction and agency at the meta-level iteratively wield their influence on lower levels, such as individuals, their perceptions, and behavioral outcomes.

Interactions between the mini- and meta-levels are “the glue that holds societies together” (Göpel, 2016, p. 47) and can be promising research topics of a transformation-oriented psychology. Here, we exemplify with two psychological theories, namely Self-Determination Theory and Self-Efficacy Theory, how psychological perspectives could be embedded in Göpel's MLP (Göpel, 2016). Figure 1 depicts these thoughts.

### Self-Determination Theory

Self-Determination Theory (Deci and Ryan, 2000; Ryan and Deci, 2017) is a humanistic, organismic-dialectical theory of human motivation. It proposes the universal, innate, basic psychological needs for autonomy (agency), competence (efficacy), and relatedness (belonging) as pre-requisites for healthy human functioning and self-sustaining, autonomous motivation. If these needs are frustrated rather than satisfied, humans become defensive, have difficulty integrating threatening information, and struggle to cope with challenges in proactive, healthy ways. Given that actors at all societal levels perceive the climate crisis and its subsequent implications for societal transformation as threatening and challenging, understanding basic psychological need satisfaction is critical (see Wullenkord, 2020).

Being a dialectical theory, Self-Determination Theory goes beyond the traditional individualistic approach of cognitive psychology and thereby fits well into transition-oriented ways of thinking. It proposes that need satisfaction is a function of the social context: Social contexts mediate in how far individuals or groups (e.g., activists in grassroots movements) can satisfy their needs. This, in turn, affects how individuals shape those contexts to be need-satisfying. For instance, the meso-level may set actual constraints in how far people *can* meet their needs (e.g., laws promoting social inequality may thwart need satisfaction), while the meta-level may influence how people *perceive* their needs to be met (e.g., narratives around growth-orientation represent need-frustrating, extrinsic values). Individual need satisfaction influences how individuals shape their proximate contexts, indirectly shaping niches and regimes, and contributing to the predominant way of understanding the world (meta-level).

### Self-Efficacy Theory

Bandura's Self-Efficacy Theory (1997) arose as a critique of Skinner's (1971) behaviorism, assuming that humans are agentic beings that have the power to shape their surroundings (see Bandura, 2019 for a summary). Thus, Self-Efficacy Theory might be a suitable framework to investigate transformations in which individuals are not only the outcome of higher-level influences but actively create those settings as political agents. Self-efficacy is the belief that one is able to perform a specific behavior to produce certain outcomes (Bandura, 1997). Previous research has mainly considered behavioral self-efficacy (i.e., the belief that one can perform certain *behaviors*, Bandura, 2006a), in contrast to outcome expectancy (i.e., the belief that an action produces certain *outcomes*, Bandura, 1997). Perceived collective efficacy (i.e., the belief that a group agent can produce certain outcomes, Bandura, 1997) seems particularly important for collective change.

Self-efficacy affects people's aspirations, accomplishments, well-being, and perseverance in goal-pursuit in the face of difficulties (Bandura, 2006b, 2019). We hypothesize that behavioral self-efficacy evolves mostly from direct feedback and experiences made on the meso- and micro-level (e.g., regime lock-ins), while outcome expectancies and collective efficacy regarding societal transformations might be more strongly mediated by meta-level influences like success stories and visions as indirect social feedback (e.g., cognitive lock-ins). Even though Self-Efficacy Theory is primarily an individual-focused social cognitive theory, it may provide a basis for investigating actual (not only perceived) collective agency (see Empowerment Theory, Cattaneo and Chapman, 2010).

## Discussion

Based on the above considerations, we suggest how environmental psychology research could become more transition-oriented and exemplify how we may change our own research practices to contribute to socio-technical transition research. When we provide examples, we mainly focus on the university regime, even though a vast array of research topics is possible.

**We need to develop and consider transformation-oriented concepts and connect them with psychological constructs and processes**. To this end, we need to engage in the discourse on transition studies, set transformation-oriented research agendas that bridge systemic and individual perspectives, and phrase research questions accordingly. In the context of needs, one may ask “How do student initiatives as exemplary niches satisfy needs and thus foster autonomous motivation for long-term engagement, constantly recreating themselves to meet the needs of their members?” Further, “What influence does students' collective efficacy have on environmental intentions?” (Hamann and Reese, 2020, study 1) could become “What influence does students' collective efficacy have on a transformation of the university regime and how does the university regime in turn influence students' collective efficacy?”

**We need to acknowledge real-life contexts as cause and consequence of individual behavior**. To do so, we need to fit our theories to the contexts in which we use them. Even though criticized as reductionist and mechanistic (see Bögel and Upham, 2018), the Theory of Planned Behavior (Ajzen, 1991) is useful in contexts in which mindful decisions are possible. Yet in most contexts, theories bridging different levels might be more appropriate (e.g., value- and identity-oriented, dialectical theories, see Bamberg, 2018, Schulte et al., 2020). To acknowledge real-life contexts, we could focus on regime- or niche-specific research questions, include more long-term perspectives, and draw on data sets that are representative for specific contexts (see Brick, 2021 for a collection of openly available, large-scale datasets). Moreover, we might apply methods from other disciplines to get a better picture of contexts. For example, to investigate students' collective efficacy in the university regime, we could examine university polls and university visions (meta-level) and collect data on university size and infrastructure (meso-level, e.g., complementing quantitative data collection with interviews). We would then not only examine individual behavioral outcomes but actual political change, changes in university narratives, participation processes, and the effectiveness of student actions.

**We need to focus more on niches**. Even though pioneer activity plays a crucial role in many transition models (e.g., Geels, 2011), it is largely underemphasized in environmental psychology. We need a discussion about niche groups, niche practices, and their respective influence (see Becker et al., 2021). For example, one may consider students' need satisfaction when participating in niches that aim to transform the university regime (e.g., install a green-office) and investigate psychological processes underlying long-term engagement.

**We need to view individuals as political agents**. Transition research has thus far mostly focused on technology acceptance and individuals as users or consumers (see Bögel and Upham, 2018; Köhler et al., 2019). By investigating individuals and groups as political agents, psychology could offer new perspectives to transition research with individual and collective levers of change. For example, studies could focus on university students as voters of a student parliament and active contributors to decisions relevant to what the cafeteria offers, instead of as mere consumers of (non)sustainable cafeteria products.

**We need to have a disciplinary discourse about the interdisciplinary position of environmental psychology**. The increasing amount of collaborative research teams (Kazdin, 2009) is a promising development and needs to be expanded (see Gifford, 2014). This development makes it even more important to discuss environmental psychology's place in research on socio-ecological transformation and necessary skills and resources connected to it at conferences, within research teams, or in theoretical articles (see Clayton et al., 2016).

**We need to set a transformation-oriented research agenda**. Large socio-ecological transformations could incorporate many new social practices (e.g., citizen participation, work time reduction, unconditional basic income). We might contribute to co-creating such protected spaces for niche practices in order to investigate them. For instance, we could set up living labs to explore how to deal with conflicting sustainability goals of various stakeholders (see Köhler et al., 2019).

**We need to constantly rediscover our own curiosity about real-world processes**. Finally, we propose to regularly question our own research in light of multidisciplinary theoretical and practical relevance, for instance by employing different, perhaps self-reflective methodological approaches.

### Conclusion

In this opinion piece, we exemplified why and how to integrate our disciplinary perspective into the broader discourse on transitions, and discussed implications for environmental psychology research. Of course, our own view is limited and subject to discussion. We hope to stimulate such discussion and encourage readers to reflect on their own research practices—with the overarching goal of understanding and promoting a socio-ecological transformation.

## Author Contributions

MW contributed the part on Self-Determination Theory. KH contributed the part on Self-Efficacy Theory. All authors contributed to the article and approved the submitted version.

## Conflict of Interest

The authors declare that the research was conducted in the absence of any commercial or financial relationships that could be construed as a potential conflict of interest.

## Footnotes

^1^https://ipu-ev.de/bildungsmaterialien/online-vorlesung/

^2^i.e., predominant ways of thinking.
